# Advancements in Preclinical Models for *NF2*-Related Schwannomatosis Research

**DOI:** 10.3390/cancers18020224

**Published:** 2026-01-11

**Authors:** Bo-Shi Zhang, Simeng Lu, Scott R. Plotkin, Lei Xu

**Affiliations:** 1 Edwin L. Steele Laboratories, Department of Radiation Oncology, Massachusetts General Hospital, Harvard Medical School, Boston, MA 02114, USA; 2 Department of Neurology and Cancer Center, Massachusetts General Hospital, Harvard Medical School, Boston, MA 02114, USA

**Keywords:** *NF2*-related schwannomatosis, mouse models, patient-derived models, organotypic models, tumor growth, hearing function, ataxia

## Abstract

*NF2*-related Schwannomatosis (*NF2*-SWN) remains a disorder with few effective treatment options. Patients develop vestibular schwannomas (VSs) on both auditory nerves, which gradually impair hearing and often result in significant communication difficulties, social withdrawal, and higher rates of depression. Progress in understanding *NF2*-SWN biology and translating discoveries into therapies has been slowed by the absence of robust animal models that faithfully reproduce both tumor behavior and the associated neurological deficits. In this review, we summarized the development of animal models that not only reproduce tumor growth in the peripheral nerve microenvironment but also reproduce tumor-induced neurological symptoms, such as hearing loss and ataxia. We further highlight the currently available organotypic models for *NF2*-SWN. Together, these systems provide an essential foundation for advancing mechanistic studies and accelerating the development of effective therapies for this devastating disorder.

## Simple Summary

*NF2*-related Schwannomatosis (*NF2*-SWN) remains a disorder with limited effective therapeutic options. Progress in basic and translational research has historically been constrained by the lack of robust preclinical models that faithfully recapitulate the neurological deficits associated with vestibular schwannoma (VS), particularly hearing loss and ataxia. Recent advances, however, have led to the development of mouse models that reproduce these functional impairments, alongside the emergence of patient-derived xenografts and organotypic culture systems. In this review, we summarize currently available in vivo and ex vivo experimental models and discuss how these platforms have advanced our understanding of *NF2*-SWN biology and facilitated the identification of potential therapeutic strategies.

## 1. Introduction

*NF2*-related Schwannomatosis (*NF2*-SWN) is an autosomal dominant disorder, most notably characterized by bilateral vestibular schwannomas (VSs)—non-malignant Schwann cell-derived tumors that arise from the eighth cranial nerve [1,2]. These lesions commonly lead to progressive, and often permanent, sensorineural hearing loss that disrupts communication and daily functioning, frequently contributing to social withdrawal and depression [3,4]. As VS enlarges, patients may also experience imbalance, facial weakness, and, in severe cases, life-threatening brainstem compression. Current clinical management relies almost exclusively on surgical resection or radiotherapy, both of which carry substantial risks, including further hearing deterioration [5]. Bevacizumab, a monoclonal antibody against vascular endothelial growth factor (VEGF)-A, is approved in the United Kingdom for *NF2*-SWN and can improve hearing or reduce tumor size in roughly one-third of patients, but responses are variable and often temporary [5,6,7]. These limitations underscore the need for deeper insight into the mechanisms driving schwannoma growth and hearing decline, as well as for the development of more effective targeted therapies.

Progress in basic and translational research has long been hampered by the scarcity of preclinical models that faithfully recapitulate the neurological deficits associated with VS, particularly hearing loss and ataxia. Recently, however, the field has advanced with the emergence of improved mouse models that mimic these functional impairments, as well as patient-derived xenografts and new organotypic culture systems. In this review, we provide an overview of currently available experimental models—including in vivo and ex vivo systems—and discuss how they have contributed to our understanding of *NF2*-SWN biology and the discovery of potential therapeutic strategies.

## 2. Genetically Engineered Mouse Models (GEMMs) of *NF2*-SWN

**Figure 1:**
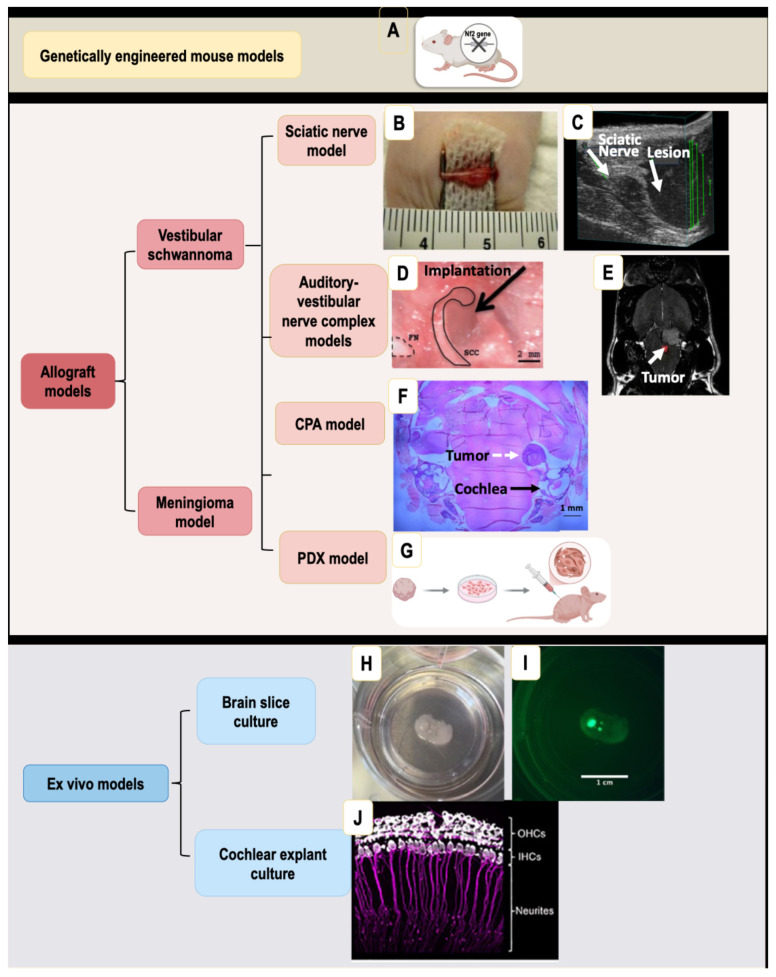
Currently available preclinical models for studying vestibular schwannomas. (**A**) Genetically engineered mouse models recapitulate *Nf2* gene loss and enable spontaneous tumor formation in vivo. Allograft models: (**B**) The sciatic nerve model, in which tumor cells are injected under the nerve sheath of the sciatic nerve to allow accessible tumor growth; (**C**) tumor formation is confirmed and can be monitored by ultrasound imaging, demonstrating compression of the sciatic nerve by the growing tumor mass. (**D**) Auditory-vestibular nerve complex models, which recapitulate tumor expansion within the internal auditory canal; (**E**) Tumor location is confirmed by magnetic resonance imaging (MRI). (**F**) Cerebellopontine angle (CPA) model, with H&E staining confirming tumor formation outside the cochlea. (**G**) Patient-derived xenograft (PDX) model, generated by implanting freshly surgically resected tumors into immunodeficient mice to preserve patient tumor heterogeneity. Ex vivo models: (**H**) Brain slice culture model, which supports tumor cell culture within the brain microenvironment; (**I**) enables mechanistic studies using immunohistological analysis. (**J**) Cochlear explant culture model, which allows investigation of tumor-hair cell–neurite interactions and tumor-induced changes in outer hair cells (OHCs), inner hair cells (IHCs), and neurites.

**Figure 2:**
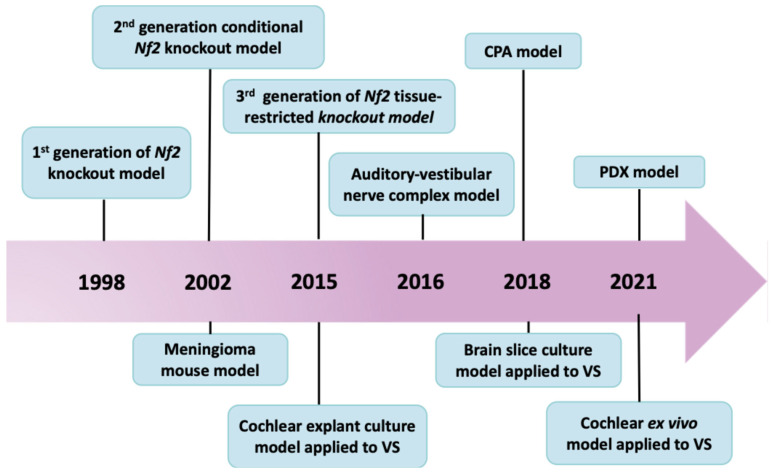
Timeline of key developments in preclinical models for vestibular schwannomas.

Several generations of *Nf2* tumor suppressor gene knockout mice have been developed. The first generation used germline heterozygous disruption of the *Nf2* gene in mice [8] (Figure 1 and Figure 2). Although *Nf2* heterozygous mice develop a variety of malignant tumors, confirming the important role of the *Nf2* gene as a tumor suppressor, they do not form schwannoma, a characteristic of *NF2*-SWN. To overcome the early embryonic lethality caused by homozygous *Nf2* loss, second-generation conditional knockout models were created using Cre/loxP strategies to delete *Nf2* in Schwann cell lineages (for example, using the P0 promoter) [9]. This model produced Schwann-cell hyperplasia and some Schwannoma formation, but did not generate tumors on the vestibular nerve. More recently, third-generation models refined *Nf2* gene excision using tissue-restricted *Cre* drivers under Periostin promoter (*Postn-Cre;Nf2^flox/flox^*), enabling mice to develop spinal, peripheral, and cranial nerve tumors histologically identical to human schwannomas, and exhibit functional impairment, such as hearing and balance deficits [10]. The strategies to generate *Nf2* knockout mice, their phenotypes, and limitations have been comprehensively reviewed [11,12], collectively illustrating the progressive evolution of GEMMs toward more accurate and clinically relevant models of *NF2*-SWN (Table 1).

## 3. Allograft Models of Schwannomas

### 3.1. Sciatic Nerve Model of Schwannomas

The first sciatic nerve model of schwannomas was developed by Chang and Welling group [13] (Figure 2). The sciatic nerve is a large, superficial peripheral nerve in the hindlimb, making it easily accessible for surgical injections (Figure 1). Tumor cells are directly injected under the nerve sheath, resulting in localized schwannomas that develop within the peripheral nerve microenvironment [14]. Tumor growth in the sciatic nerve model can be easily monitored using caliper measurement, bioluminescence imaging, or ultrasound [15,16,17].

#### 3.1.1. Utility of the Sciatic Nerve Model in *NF2*-SWN Research

Using this model, a number of studies reporting the identification of therapeutic strategies (i) targeting the schwannoma cells, (ii) targeting the tumor microenvironment, and (iii) gene therapy have been identified.

##### Targeting the Tumor Cells

cMET, a proto-oncogene involved in tumor progression, invasion, and treatment resistance, is aberrantly activated in schwannomas [18]. Crizotinib, a small-molecule inhibitor of cMET and other receptor tyrosine kinases, is approved by the U.S. FDA for ALK- and ROS1-positive non-small cell lung cancer patients. Kissil’s group showed that crizotinib reduces *NF2*-null Schwann cell proliferation in vitro and suppresses Schwannoma growth in vivo. They further identified Focal Adhesion Kinase 1 (FAK1) inhibition as a key downstream mechanism of action [19]. Using the sciatic nerve model, Fernandez-Valle’s group tested a dual-targeting strategy combining cabozantinib (a c-MET inhibitor) with saracatinib (a Src inhibitor). Src-family kinases, which regulate cell survival, proliferation, and angiogenesis, are frequently activated in schwannomas due to the loss of the *NF2* tumor suppressor gene [20]. It was demonstrated that the combination of cabozantinib and saracatinib synergistically induces Schwannoma cell apoptosis and suppresses tumor progression [21]. The mechanistic target of rapamycin (mTOR) pathway is abnormally activated in *NF*-deficient tumors. Work from Stankovic, Ramesh, and Xu’s group showed that dual inhibition of mTOR and Src pathways significantly reduces tumor growth in the sciatic nerve model, providing a strong rationale for combinatorial targeting of these convergent pathways [22].

##### Targeting the Tumor Microenvironment

VEGF is a key pro-angiogenic cytokine that drives endothelial proliferation, vascular permeability, and abnormal neovascularization in tumors [23]. In the sciatic nerve model, anti-VEGF treatment significantly inhibited schwannoma growth by reducing tumor angiogenesis and inducing cell death [15]. These findings led to the successful clinical application of bevacizumab, a humanized monoclonal antibody that neutralizes VEGF-A, in patients with *NF2*-SWN [5]. Since then, multiple clinical studies have reported that bevacizumab improves hearing and shrinks tumors in 30–40% of patients, with hearing and tumor stability in another 30–40% [5,6,7]. Bevacizumab is now approved in the UK for the treatment of patients with *NF2*-SWN and recommended by the European Association of Neuro-Oncology [24]. In addition to tumor burden, the sciatic nerve model also allows direct evaluation of neurological deficits, including motor function, coordination, and balance [17]. Xu’s group leveraged this capability to demonstrate that anti-VEGF therapy not only suppresses schwannoma growth but also promotes functional recovery by normalizing the tumor vasculature, decreasing muscle atrophy and increasing nerve regeneration in the sciatic nerve model [17,25].

##### Gene Therapy

The sciatic nerve model is also a robust platform for testing gene therapy approaches for *NF2*-SWN, it allows researchers to directly deliver gene therapies intratumorally. Brenner’s group cloned an adeno-associated virus (AAV) vector encoding pro-caspase-1 under a Schwann cell-specific promoter (P0), and directly injected the AAV into the sciatic nerve schwannomas. This study provided the first proof-of-concept that gene therapy can selectively kill schwannoma cells in vivo [26]. Building on this strategy, they further demonstrated that AAV-mediated delivery of Gasermin-D, an executor of pyroptotic cell death, also driven by the P0 promoter, markedly increased tumor cell death and significantly inhibited the growth of sciatic nerve schwannomas [27]. These studies show that schwannomas are highly vulnerable to targeted cytotoxic gene delivery, and that cell-death-based gene therapy represents a promising therapeutic modality for *NF2*-SWN. Most recently, Breakefield’s group tested a gene replacement strategy using an AAV vector expressing the full-length *NF2* tumor suppressor gene. They demonstrated that AAV-mediated delivery of functional merlin inhibits mTORC1 activation in cultured Schwann cells and that a single intratumoral injection of an AAV-merlin leads to regression of sciatic nerve tumors in vivo [28]. This study provides proof of principle that direct Merlin replacement can reverse established Schwannomas.

#### 3.1.2. Limitations of the Sciatic Nerve Model

The sciatic nerve model does not model the hearing loss, vestibular deficits, and cranial neuropathies seen in *NF2*-SWN patients. Functional endpoints are limited to tumor growth and motor function evaluation, and do not address critical issues in improving patient quality of life in *NF2*-SWN therapy development.

In conclusion, the sciatic nerve model is a powerful translational platform; it provides a rapid, cost-effective way to evaluate therapeutic agents for *NF2*-SWN. However, it does not replicate the central nervous system environment or auditory/vestibular dysfunction seen in *NF2*-SWN. Findings should be validated in more clinically relevant intracranial models to ensure translational relevance, especially for therapies targeting vestibular schwannomas (Table 1).

### 3.2. Auditory-Vestibular Nerve Complex Models

Two independent groups have established models in which Schwannoma cells are implanted directly into the auditory-vestibular nerve complex. Bonne et al. injected Schwann cells derived from *Nf2* mutant mice into the vestibular nerve region of the inner auditory canal in SCID mice [29]. Dinh et al. implanted human Merlin-deficient Schwann cells into the cochleovestibular nerve of immunodeficient Rowett nude rats [30]. Importantly, both models successfully recapitulated hearing loss induced by schwannomas [29,30,31] (Figure 1 and Figure 2).

*Limitations of the auditory-vestibular nerve complex model:* In the mouse model, direct injection of tumor cells into the auditory-vestibular nerve complex successfully recapitulates tumor growth within the inner auditory canal and results in hearing loss. However, the surgical approach itself—the sham injection—immediately caused hearing loss, and animals may require up to 14 days to recover baseline hearing function. This procedure-related hearing loss complicates the interpretation of early post-implantation auditory outcomes.

In the rat model, the larger skull size substantially facilitates surgical manipulation and improves procedure precision. Nevertheless, the limited availability of inbred rat strains and the scarcity of well-characterized rat schwannoma cell lines constrain the use of this allograft approach. Although immunodeficient rat strains are available, they are far fewer than their murine counterparts, restricting the feasibility of xenograft studies using patient-derived schwannoma cells.

In conclusion, both models generate tumors in anatomically relevant locations and produce measurable hearing loss, validating their utility for investigating the mechanisms of vestibular schwannoma-associated auditory dysfunction and for preclinical therapeutic testing. However, their technical complexity and limited scalability reduce their adaptability for broader preclinical applications (Table 1).

### 3.3. Cerebellopontine Angle (CPA) Model of Vestibular Schwannoma

Vestibular schwannomas arise at the CPA near the vestibulocochlear nerve (cranial nerve VIII). The CPA model was established by the Xu L group [32] (Figure 1 and Figure 2). In this model, tumor cells were stereotactically injected into the mouse CPA region, formation of a tumor in proximity to the cochlear nerve was confirmed by magnetic resonance imaging (MRI), and tumor-induced hearing loss was evaluated using auditory brainstem response (ABR) and Distortion Product Otoacoustic Emissions (DPOAE).

#### 3.3.1. Utility of the CPA Model in *NF2*-SWN Research

The CPA model allows tumor implantation near cranial nerves, mimicking the tumor microenvironment of intracranial vestibular schwannomas. The CPA is a confined space involving the brainstem, cranial nerves, and cerebellum, allowing the model to reproduce tumor mechanical compression and brainstem displacement, as seen in human pathology. In the CPA model, tumor growth leads to hearing loss. Notably, tumor size does not correlate with the severity of hearing loss, faithfully recapitulating the clinical situation in human patients, in whom VS tumor size similarly does not correlate with hearing impairment.

##### Targeting the Tumor Cells

Using the CPA Model, strategies targeting tumor cells and the tumor microenvironment have been implemented. Targeting tumor cell cMET signaling, it was found that cMET blockade with crizotinib enhances tumor radiosensitivity by increasing DNA damage, and combined crizotinib treatment improved radiation therapy efficacy in the CPA model. Importantly, cMET blockade itself does not cause ototoxicity and can protect against it in the context of radiation therapy [33]. Currently, a Phase 2 clinical trial is underway to evaluate the effectiveness and safety of crizotinib in children and adults with *NF2*-SWN and progressive vestibular schwannomas (NCT04283669).

##### Normalizing Tumor Microenvironment

Using mouse cell lines and human patient samples in the CPA model, it was demonstrated that losartan, an FDA-approved antihypertensive drug, is a potential therapeutic agent for managing tumor-induced hearing loss. Losartan treatment, by inhibiting the fibrogenic and inflammatory angiotensin signaling pathway, normalized the tumor microenvironment and reduced neuroinflammation and neuro-edema, thereby alleviating tumor-induced hearing loss. More importantly, the preclinical study is consistent with retrospective analysis of patients with both VS and hypertension, which revealed that those treated with losartan experienced no progression in hearing loss, unlike patients on other or no antihypertensive medications [34]. Currently, Massachusetts General Hospital is planning a Phase 2 clinical trial to assess the effects of losartan on hearing preservation.

##### Alleviating VS-induced Ataxia

In Addition to tumor burden and animal survival, the CPA model also provides an important platform for studying vestibular dysfunction. Patients with bilateral VS often sustain damage to the vestibular nerve or vestibule apparatus [35], resulting in debilitating symptoms, such as impaired balance, ataxia, and muscle weakness [36]. Mice bearing tumors in the CPA region develop symptoms of ataxia and incoordination. Lu et al. established a panel of tests to evaluate the mouse gait, coordination, and motor function [37]. This model, therefore, provides an opportunity to study tumor-induced neurological deficits and to evaluate the effects of therapeutic intervention on vestibular function.

#### 3.3.2. Limitations of the CPA Model

Tumor implantation into the CPA is technically demanding and requires stereotactic precision. Due to the confined anatomical space, tumors may rapidly compress the cerebellum and brainstem, necessitating early sacrifice of animals, which restricts the ability to evaluate long-term tumor progression and chronic treatment effects. Brain size varies across species and with age; therefore, the injection location needs to be carefully calibrated for different species and age cohorts.

In conclusion, the CPA model is a more anatomically relevant preclinical model for *NF2*-SWN than the peripheral nerve sciatic nerve model, and provides valuable anatomical and functional relevance for *NF2*-SWN research, particularly for evaluating treatment effects on hearing and the severity of ataxia. However, because of the confined anatomical space and proximity to vital structures, the CPA model is limited by surgical complexities and the risk of complications arising from tumor compression (Table 1).

### 3.4. Patient-Derived Xenograft (PDX) Model

PDX models, in which tumor fragments surgically dissected from *NF2*-SWN patients are directly implanted into immunodeficient mice, have emerged as a useful model for translational research aimed at facilitating precision medicine and drug development. While malignant cancer cells can often grow indefinitely in cell culture and readily form xenograft tumors in mice, establishing PDX models for VS has been challenging because vestibular schwannomas are non-malignant and slow-growing. Consequently, only a limited number of *NF2*-SWN PDX models have been reported (Figure 2).

Two recent studies have advanced the development of PDX models for *NF2*-SWN. Wu et al. established human vestibular schwannoma cell lines using SV40-mediated immortalization and implanted these cells into the CPA of nude mice [34]. Zhao et al. directly implanted surgical specimens from patients with *NF2*-SWN subcutaneously into NOD/SCID mice, followed by serial passaging to select for stable engraftment and growth characteristics [38] (Figure 1).

#### 3.4.1. Utility of the PDX Model in *NF2*-SWN Research

Unlike murine tumor models, PDXs preserve the histological features and genetic heterogeneity of patient tumors, providing a more clinically relevant platform for translational studies and the development of personalized therapies.

Using this approach, Wu et al. elucidated the role of tumor extracellular matrix in mediating hearing loss and treatment response. They showed that losartan, an FDA-approved anti-hypertensive that blocks angiotensin signaling, reduced tumor fibrosis and normalized tumor vasculature and oxygen delivery. This tumor microenvironment (TME) reprogramming enhanced the efficacy of radiation therapy, enabling effective tumor control at a lower radiation dose. Importantly, losartan treatment also inhibited neuroinflammatory IL-6/STAT3 signaling in tumor-associated macrophages, thereby preventing tumor-induced hearing loss.

Zhao et al. leveraged their PDX biobank for high-throughput drug screening and identified potent PI3K/AKT/mTOR inhibitors, including AZD8055 and PQR309, which effectively suppressed tumor growth both in vitro and in PDX models [38].

#### 3.4.2. Limitations of the PDX Model

First, as Schwannoma PDX models are established in immunodeficient mice (e.g., NSG or nude strains), they share the inherent limitations associated with PDX models. Although the implanted tumor cells are of human origin, the stromal compartment, including vascular endothelial cells and inflammatory and immune cells, remains murine. This interspecies mismatch may prevent full recapitulation of the human tumor microenvironment. More critically, the absence of a functional immune system precludes proper evaluation of tumor-immune interactions, thereby limiting the utility of PDX models for studying immunological mechanisms or for preclinical testing of immunotherapies relevant to *NF2*-SWN. Humanized mice, which more closely recapitulate human immune components, represent an ideal host for patient-derived xenograft models. However, their high cost makes them impractical for large-scale drug development studies. To assess the contribution of the immune components to VS progression and neurological dysfunction, a small cohort of humanized mice can be used to compare tumor growth, hearing loss, and neurological function in VS models established in nude mice vs. in humanized mice.

Second, as a rare disease, patient-derived *NF2*-SWN PDX models remain limited. To more accurately capture patient heterogeneity and improve the robustness of preclinical analyses, it will be essential to establish a multi-institutional biobank of patient-derived schwannoma cells or xenografts collected from multiple hospitals and research centers. Such collaborative efforts would increase sample diversity, enhance statistical power, and enable more representative modeling of *NF2*-SWN biology across the spectrum of genetic mutations and clinical presentations.

Third, because schwannomas are non-malignant and intrinsically slow-growing, PDX models often exhibit low tumor take rates, making it challenging to establish stable, reproducible PDX lines. Several approaches have been applied to improve tumor take, including in vivo selection of more proliferative tumors through serial passaging and co-implantation with supportive stromal components, such as macrophages or fibroblasts, to support early tumor survival. Additional strategies under investigation include optimizing implantation sites by implanting intramuscularly, where the abundant vascular supply may enhance engraftment and growth. Nevertheless, given the benign biological nature of schwannomas, low engraftment efficiency is likely to remain an inherent limitation of PDX models, underscoring the need to complement these systems with alternative preclinical platforms.

Lastly, the lack of a standardized protocol for establishing a PDX model may introduce variability among models generated by different institutions. While one study reported successful PDX generation from untransformed primary cells [34], other reports indicate that cell immortalization—typically achieved through SV40 or hTERT infection—is required for stable propagation [38]. These immortalization methods, however, differ markedly in their biological effects. SV40 promotes immortalization by inactivating tumor suppressor pathways such as p53. SV40 can cause tumor growth on its own and disrupts DNA damage responses, introducing significant genetic alterations. In contrast, hTERT-mediated immortalization prevents telomere shortening while essentially maintaining normal DNA repair capacity and karyotypic stability, thus producing cells with fewer transformation-associated artifacts. Future studies that systematically compare the impact of different immortalization strategies on the biological and tumorigenic properties of *NF2*-SWN–derived cells will be essential to develop reliable and standardized models for use in PDX and other preclinical platforms. Such efforts will ultimately improve reproducibility and facilitate meaningful cross-study comparisons in schwannoma research.

In conclusion, PDX models of *NF2*-SWN are a promising tool for studying patient-specific tumor biology and for preclinical drug screening. However, they are constrained by low engraftment rates, a paucity of immune components, and practical challenges in establishing models (Table 1). Their greatest value may lie in combination with other models (e.g., GEMMs, organoids) to provide a comprehensive understanding of *NF2*-SWN pathogenesis and treatment response.

### 3.5. Meningioma Model

Meningiomas occur in approximately 80% of patients with *NF2*-SWN during their life eventurally and often present as multiple intracranial tumors, contributing substantially to morbidity and mortality [39]. Xenograft models of meningioma have been developed using human tumor cells, including established cell lines and patient-derived primary cells. In *NF2*-associated meningioma models, primary tumor cells or the IOMM-Lee cell line are stereotactically injected into immunodeficient mice at intracranial sites, such as the subdural space over the cerebral convexity or the skull base dura. Tumor growth is subsequently monitored using histological analysis or non-invasive luciferase-based bioluminescence imaging [40].

A recent advance established a novel spontaneous meningioma mouse model using Cas9-based genome editing to induce targeted inactivation of four tumor suppressor genes—*Nf2*, *P15^Ink4b^*, *P16^Ink4a^*, and *P19^Arf^*— in meningeal cells of neonatal transgenic mice [41]. Following AAV-Cre/gRNA delivery to the skull-base meninges and longitudinal observation for up to 10 months, a substantial fraction of mice developed intracranial tumors that were morphologically and immunohistochemically consistent with human meningioma [41]. This model enables meningioma development from normal meningeal cells in vivo, providing a genetically faithful platform for studying tumor initiation and progression (Figure 1 and Figure 2).

#### 3.5.1. Utility of the Meningioma Models

Using these preclinical models, Ramesh, Chang, and colleagues have identified several signaling pathways critical for *NF2*-deficient meningioma proliferation, motility, and survival, including group I p21-activated kinases (PAKs), EPH receptor and Src-family kinases, mTOR signaling, and histone deacetylase [42,43,44,45]. Pharmacological targeting of these pathways—using PAK inhibitors (FRAX597, FRAX716, FRAX1036), tyrosine kinase inhibitors (brigatinib), histone deacetylase inhibitors (AR-42), or third-generation mTORC1 inhibitors (RMC-6272)—suppressed tumor growth, induced apoptosis, and modulated cell-cycle regulators in preclinical studies [42,44,45,46,47].

Additionally, Nigim et al. stereotactically implanted malignant meningioma cells into the frontal subdural space of immunodeficient mice, and demonstrated that intratumoral administration of oncolytic herpes simplex virus G47Δ significantly reduced tumor burden and prolonged survival compared with vehicle-treated controls [48]. Similarly, Bao et al. reported that ferroptosis-inducing therapy using Erastin in orthotopic models of *MEF2C*-knockdown meningioma cells reduced tumor volume and extended survival, identifying *MEF2C* as a key modulator of ferroptosis sensitivity in *NF2*-deficient meningiomas [49].

#### 3.5.2. Limitations of the Meningioma Model

Human meningiomas arise from the dura mater at diverse anatomical locations, including the cerebral convexity, the skull base, and the spinal dura. However, mouse models that faithfully recapitulate tumors arising from the spinal dura or deep, anatomically complex skull base regions remain limited, thereby restricting the ability to fully model the spatial and microenvironmental complexity of human disease. Moreover, human meningiomas typically grow as dural-attached, encapsulated lesions that may invade adjacent bone and brain tissue, whereas xenograft tumors often form discrete, mass-like lesions lacking natural encapsulation and true invasive behavior.

Collectively, these limitations indicate that meningioma xenograft and PDX models are suboptimal for mechanistic studies of tumorigenesis and local invasion. Nevertheless, they remain highly valuable for preclinical therapeutic testing.

In conclusion, Meningioma xenograft and PDX models remain the most widely used platforms in preclinical meningioma research and are well-suited for therapeutic evaluation. However, their limitations in modeling tumor microenvironment, anatomical origin, and disease initiation underscore the importance of integrating these systems with genetic and spontaneous models to achieve a more comprehensive understanding of *NF2*-associated meningioma biology and treatment response (Table 1).

### 3.6. Future Directions of Allograft Models for NF2-SWN

Although substantial progress has been made in developing preclinical models for VS, several aspects of current allograft models remain to be optimized.

First, all existing allograft models rely on *NF2*-wildtype host mice. Tumor-host interactions are well documented to play a critical role in tumor progression, immune response, and therapeutic efficacy. Therefore, implanting VS tumor cells in *NF2*-deficient host mice would more faithfully model the contribution of *NF2* gene loss to VS tumor growth and treatment response. However, this will require breeding the *Postn-Cre;Nf2^flox/flox^* mouse into an immunodeficient background for the PDX model and breeding into the C57B/L6-FVB background for the syngeneic models.

Second, biological variability related to mouse sex and age may significantly influence experimental outcomes, particularly for functional endpoints, such as hearing and ataxia. Current allograft studies typically use young adult mice (8–12 weeks of age), which may not adequately capture age-dependent susceptibility to hearing loss or neurological dysfunction. Systematic evaluation of age-related differences in hearing loss and ataxia in VS models will be essential to improve the reproducibility and translational relevance of allograft models of *NF2*-SWN.

## 4. Ex Vivo Models

Organotypic culture systems have emerged as a powerful tool in neurofibromatosis research, providing a physiologically relevant platform to study tumor–nerve–immune cell interactions in a controlled ex vivo environment. By preserving the three-dimensional architecture and cellular heterogeneity, organotypic cultures enable detailed mechanistic studies of Schwann cell tumor growth, neuronal crosstalk, and microenvironmental remodeling that are difficult to recapitulate in dissociated cell culture systems. Furthermore, organotypic models allow real-time imaging, pharmacological perturbation, and genetic manipulation, making them invaluable for preclinical testing of candidate therapies aimed at modulating tumor microenvironment, neuroinflammation, and tumor progression while reducing reliance on in vivo models.

### 4.1. Brain Slice Culture Model

Brain slice culture is an ex vivo technique that enables patient-derived tissue to grow within an intact neuronal microenvironment, preserving the native cytoarchitecture, synaptic connectivity, and cellular heterogeneity of the brain. In *NF2*-SWN research, brain slice cultures—particularly from cerebellum and brainstem regions where vestibular schwannomas arise—provide essential microenvironmental support that these slow-growing, non-malignant tumors cannot achieve in conventional 2D in vitro culture.

#### 4.1.1. Utility of Brain Slice Culture in *NF2*-SWN Research

Zhao et al. used an organotypic brain slice culture system to grow fresh patient-derived vestibular schwannoma and meningioma samples and evaluate their response to the cMET inhibitor, crizotinib [33] (Figure 1 and Figure 2). In their study, CPA regions from the mouse brain were sectioned into 300 μm slices and maintained in culture. Fresh surgical tumor specimens were cut into ~1 × 1 mm fragments and implanted onto the brain slices. After two days of engraftment, crizotinib was added to the medium, and the brain slices were cultured for up to two weeks before fixation and histological analysis. The authors reported that cMET blockade suppressed tumor growth, reduced tumor cell cMET signaling, and decreased tumor cell proliferation.

#### 4.1.2. Limitations of Brain Slice Culture

Like any organotypic culture, brain slice culture has a relatively short viability window—brain slices typically remain healthy for only 1–3 weeks, limiting the ability to study long-term tumor behavior. It also lacks key components, such as a blood supply and immune cell infiltration, reducing the ability to model whole-organism treatment responses (Table 1).

In conclusion, brain slice cultures preserve the critical cell–cell and cell–matrix interactions that are absent in 2D culture systems, providing essential microenvironmental support for slow-growing, non-malignant schwannoma patient samples. This platform is well-suited for live imaging and electrophysiological studies to investigate tumor-neuron, tumor-microglia, and tumor-matrix interactions. Most importantly, brain slice cultures enable drug testing directly on patient-derived tumors, supporting the development of personalized therapeutic strategies. Together, these advantages position brain slice culture as a powerful tool to bridge molecular mechanisms to functional outcomes in *NF2*-SWN pathogenesis and therapy development.

### 4.2. Cochlear Explant Culture Model

Cochlear explant culture is a robust ex vivo model that preserves the cytoarchitecture of the organ of Corti, spiral ganglion neurons (SGNs), and supporting cells, enabling detailed studies of auditory biology and otic pathology under controlled conditions. In the context of *NF2*-SWN, where vestibular schwannomas damage hearing through both mechanical compression and secreted ototoxic factors, cochlear explant cultures provide a powerful system to dissect the mechanisms of tumor-induced cochlear damage (Figure 1 and Figure 2).

#### 4.2.1. Utility of Cochlear Explant Culture in *NF2*-SWN Research

Cochlear explant culture preserves the entire cochlear turn, including the organ of Corti and SGN somata, allowing direct visualization of tumor-induced cochlear pathology [50]. In *NF2*-SWN, this model has been instrumental in elucidating mechanisms of tumor-mediated cochlear damage. Although sensorineural hearing loss (SNHL) in *NF2* has traditionally been attributed to mechanical compression by the tumor, multiple clinical studies have shown that tumor size and intracanalicular extension do not reliably correlate with hearing thresholds [51]. These observations have led to the emerging hypothesis that tumor-secreted factors directly contribute to cochlear injury—an idea that can be rigorously tested using cochlear explant cultures.

Dilwali et al. used the mouse cochlear explant model to study the effects of tumor secretomes on hair cell. In their study, conditioned media derived from freshly resected sporadic VS specimens were collected and added to mouse cochlear. VS tumor secretomes induced diverse cochlear injuries, including hair cell loss and SGN neurite degeneration. Notably, injury severity increased from apical to basal cochlear turns and correlated with patients’ degree of SNHL. The authors further identified tumor necrosis factor-alpha (TNFα) as a candidate ototoxic factor and fibroblast growth factor-2 (FGF2) as a potential otoprotective factor, providing early mechanistic evidence that VS-secreted molecules can either damage or protect cochlear structures [52].

##### Expanding on This Concept

Wu et al. analyzed conditioned media from 57 VS specimens obtained from patients with varying hearing loss. They demonstrated a significant negative correlation between IL-6 concentrations in VS-conditioned media and hair cell viability in cochlear explants. Exposure to high–IL-6 media resulted in pronounced outer hair cell loss and basal turn neurite degeneration, providing direct mechanistic evidence that inflammatory cytokines secreted by VS tumors contribute to cochlear injury and hearing decline in patients with *NF2*-SWN [34].

#### 4.2.2. Limitations of Cochlear Explant Culture

Despite its strengths, the cochlear explant model has important limitations. First, explants are typically derived from neonate mice, whose structure and functional maturation differ from those of adult human cochleae, potentially limiting direct translational relevance to patients with *NF2*-SWN. Second, explants have a limited in vitro lifespan—generally ranging from several days to one week—making them unsuitable for studying chronic or long-term ototoxic processes associated with persistent tumors. Third, the technique requires substantial technical expertise, as key procedural steps, such as removal of the delicate tectorial membrane, demand extensive training to achieve consistent and reproducible results [50] (Table 1).

In conclusion, cochlear explant culture enables direct exposure of cochlear tissue to tumor-conditioned media, cytokines, or pharmacological agents, allowing mechanistic interrogation of hair cell loss, synaptic degeneration, and neuronal dysfunction. Importantly, this platform supports real-time imaging, electrophysiological assessment of auditory-nerve activity, and quantitative analysis of ribbon-synapse integrity, providing functional readouts that complement in vivo hearing tests. By facilitating screening of otoprotective agents, the cochlear explant model serves as a critical bridge between mechanistic discovery and translational strategies aimed at preserving hearing in patients with *NF2*-SWN.

## 5. Conclusions

The development of a diverse array of preclinical models has substantially advanced our understanding of *NF2*-SWN and its associated hearing loss. GEMMs faithfully recapitulate schwannoma initiation, providing critical mechanistic insight into *NF2*-driven tumorigenesis. Orthotopic in vivo models—including sciatic nerve, auditory-vestibular nerve complex, and CPA models—offer anatomically and physiologically relevant platforms for evaluating tumor growth, tumor microenvironmental interactions, and therapeutic response. Patient-derived xenografts preserve tumor heterogeneity and genetic fidelity, supporting the development of precision therapeutic strategies. Complementary ex vivo organotypic systems, including brain slice and cochlear explant cultures, enable high-resolution investigation of tumor–nerve interactions, inflammatory signaling, and ototoxic mechanisms, while facilitating pharmacological screening. Together, these preclinical platforms provide a comprehensive framework for dissecting *NF2*-SWN pathophysiology, evaluating candidate therapies, and accelerating translational efforts to preserve neurological function and improve patient outcomes (Table 1).

## Figures and Tables

**Table 1:** Preclinical models of *NF2*-SWN.

Models	Advantages	Limitations
GEMM	Recapitulate *NF2* gene mutations	Slow and variable tumor development Does not model hearing loss Complex breeding schemes hinder drug testing
**Allograft models**		
Sciatic nerve model	Easy tumor access to monitor tumor growth Allows evaluation of motor function	Does not model intracranial microenvironment Does not model hearing loss
Auditory-vestibular nerve models	Recapitulate hearing loss and vestibular dysfunction	Surgical complexity Implantation-related hearing loss
CPA model	Tumor implanted in the correct anatomic location Reproduce tumor-induced hearing loss Reproduce tumor-induced ataxia	Technical difficulty
PDX model	Preserves patient genetic mutation	Limited patient-derived cell lines Low engraftment rate
Meningioma model	Suitable for therapeutic testing	Limited modeling of dural origin
**Ex vivo models**		
Brain slice model	Preserves brain microenvironment	Lacks vasculature Relatively short culture duration
Cochlear explant model	Enables investigation of direct tumor effect on cochlear	Requires neonatal tissues Limited culture duration

## Data Availability

This study did not create or analyze any new data. Data sharing is not applicable to this review article.
